# The single and interactive effects of aluminium and low pH, or Ca/Al ratios on red pine seedlings

**DOI:** 10.1186/s13104-023-06609-3

**Published:** 2023-11-17

**Authors:** Yunfeng Shan, Ammara Riaz

**Affiliations:** 1 College of Mathematics and Computer Sciences, Guangxi Science and Technology Normal University, Laibin, 546199 China; 2 iREG, Toronto, ON M5S 2C6 Canada; 3 Department of Life Sciences, Khwaja Fareed University of Engineering and Information Technology, Rahim Yar Khan, 64200 Pakistan; 4 United Graduate School, Tokyo University of Agriculture and Technology, Fuchu, Tokyo, 183 Japan

**Keywords:** Solution culture, High aluminum concentration, Low pH, Ca/Al molar ratio, Aluminum phytotoxicity, Red pine

## Abstract

Acid deposition has been one of the major environmental pollution problems for longer than 50 years, since the 1970s. Investigation on the interactive effects of aluminum (Al) and low pH or Ca/Al ratios on red pine (*Pinus densiflora* Sieb. and Zucc.) has been required but lacking. In the present study, needles of red pine seedlings exposed to Al treatments with solution pH 4.0 and 3.5 exhibited purplish leaf characteristics of Al toxicity. The dry weights of the needle and whole plant, and the current needle elongation were linearly reduced with Al concentrations from 0, 13 to 26 ppm. Results show that red pine is an intermediate species in sensitivity to Al and is insensitive to low pH. However, the synergistic interactions of low pH treatments with the elevated Al were significant. Al toxicity to red pine was significantly enlarged with reduced pH. The root length and whole plant length were significantly decreased at 1:10 of Ca/Al ratios (p < 0.05), but Al phytotoxicity was completely lost when the Ca/Al molar ratio was 10:1. Liming is still an applicable measure to remediate acidification problems by natural or anthropogenic factors such as acid deposition.

## Introduction

Soil acidification is caused by natural processes and man-made factors such as acid mine drainage, metalliferous and non-metalliferous ores near coal combustion power plants and other industrial facilities, and acid deposition has induced many degraded lands and forest declines in recent decades. Soil solution aluminum (Al) concentrations increase exponentially with increases in hydrogen ion concentration below 5.5 [1]. It is well established that high Al concentrations can damage plants [2–9]. Al toxicity was considered a cause of forest decline [10]. Effects of Al and acid deposition on tree growth have risen with the concern of forest decline in Europe, North America, and Eastern Asia [11, 12]. Acid deposition has been one of the major environmental pollution problems for longer than 50 years, since the 1970s. Acid deposition has deteriorated since 2008 in southern China [13]. Having analyzed the sum of the rates of all processes plus free acidity (H^+^) flux in throughfall that constitutes total proton load (TPL) to a soil, rates of internal net proton load (INP) and TPL were evaluated for six experimental forests in northwestern Germany after they had been monitored in long-term studies. Although internal natural sources are relevant contributors to total proton load, H^+^ load due to atmospheric deposition exceeds 70% of TPL at five of the six sites [14, 15]. As a result, acid deposition inputs much more protons into the soil than natural processes, further, accelerates the acidification of acidic soil in tropical and subtropical areas, and exacerbates the problems related to acidic, aluminum-rich soil [15]. The damages of acidification and Al toxicity to crops and forests have been a significant problem [2–5, 8, 9]. However, little relevant research was conducted regarding the single and interactive effect of low pH and elevated Al on red pine although they co-occur on Earth. The red pine is widely distributed and cultured in Japan and China. Red pine forest decline in Japan was reported [16–18]. Soil acidification reduced the growth of red pine seedlings, and soil analysis showed that the water-soluble Al concentrations were elevated with soil pH reduction [19]. Trees are different in sensitivity to Al or low pH [20]. However, investigation on the interaction of Al and low pH effects on red pine has been lacking even though both cooccur in environments. Therefore, it is required to study the interactive effects of low pH, Al toxicity, and calcium (Ca) on red pine to determine the causes of red pine forest decline, the remediation measures, and re-vegetation in these damaged lands. Toxicity due to low pH or high Al concentration *per s*e is difficult to investigate because of complex soil chemistry and lack of good understanding of ion uptake of roots in any case. In solution culture, because the concentrations of Al and other elements can be controlled, the single and interactive effects of Al and low pH or Al and Ca/Al ratio can be examined. The present study was therefore undertaken to determine single and interactive effects of Al and low pH or Al and Ca/Al ratio on the growth of red pine (*Pinus densiflora* Sieb. and Zucc.) seedlings through a solution culture experiment.

Aluminum toxicity is a major factor limiting crop and forest productivity [3, 4, 8, 9]. Farmers often apply lime to raise soil pH and thus reduce dissolved Al to safe levels and raise the Al/Ca ratio in soils. The whole plant dry weights of red pine seedlings were significantly increased with reduced pH of soil acidification treatments of pH 5.90, pH 4.65, and pH 4.10 [16], while the effects of soil acidification treatments of pH 4.6, pH 3.9, and 3.6 on whole plant dry weights were no significant [16]. Soil analysis showed that the Al concentrations were elevated. At the same time, Ca and Mg concentrations were also significantly increased in soil solution with reduced pH of soil acidification treatments. As a result, the Al/Ca molar ratios in soil were very high (< 0.09) [16]. Therefore, it is required to investigate whether the toxicity of Al to red pine seedlings can be reduced by the elevated Ca concentrations or Ca/Al ratios and whether the addition of lime is still an applicable measure for the remediation of acidification caused by acid rain using solution culture experiments.

## Materials and methods

### Al and pH treatments

Nutrient solution cultures: Seeds of red pine were obtained from a seedling nursery in Tokyo. Seeds were sown in a pot (40 cm diameter) filled with red-yellow soil from the surface forest soil. When seedlings were about 6 cm high, they were transplanted into plastic containers (15.8 cm diameter, 19.0 cm height, and 3 L size) filled with nutrient solution. Nutrients were supplied to the seedlings using a modified Saito's solution [21], the modification being to replace inorganic Ferrum (Fe) with Fe-EDTA, as shown in Table 1. Al was added as AlCl_3_. Al concentrations used were 0, 13, and 26 ppm. NaOH or HCl was used to adjust the solution pH to 4.5, 4.0, and 3.5. Therefore, a 3 × 3 factorial design was employed. Each pot was aerated by an air pump. There were ten seedlings per pot and nine pots total. Seedlings were grown for 4 weeks in a glasshouse. Nutrient solutions were replaced once every 5 days. The pH was also adjusted at the mid-point of each 5 days.

**Table 1:** Salts and elements concentrations in nutrient solution

Salts	Concentrations (mg/L)	Elements	Concentrations (mg/L)
NH_4_NO_3_	114.3	N	40
KH_2_PO_4_	38.3	p	9
K_2_SO_4_	49.5	K	33
CaCl_2_.2H_2_O	52.3	Ca	14
MgSO_4_.7H_2_O	61.2	Mg	12
MgCl_2_.6H_2_O	50.4	Fe	3
Fe-EDTA	26.4	N/A	N/A

### Ca/Al molar ratio treatments:

The solution culture method was the same as stated above in Sect. "Al and pH treatments" (Saito, 1977). Ca concentrations varied with Al concentration and designed Ca/Al molar ratios (10, 1, 0.1). Al and Ca were added as AlCl_3_ and CaCl_2_^.^ 2H_2_O, respectively. The Al concentration used was 26 ppm. NaOH or HCl was used to adjust the solution pH to 3.50 for all the treatments. Ca concentrations in solution culture without Al were 0.3, 3, and 30 mM. There were ten seedlings per pot. The seedlings were grown for 6 weeks in a glasshouse.

### Measurements

At the end of the experiment, the needle length, whole plant length, and fresh weights of whole plants were measured. At harvest, roots, stems, and leaves were rinsed in deionized water, dried at 80 ℃ for a week, and weighed.

### Statistical analysis statistical analysis

All results are from a single representative experiment with ten plants in each pot. Data were analyzed by analysis of variance (ANOVA) as a 3 × 3 factorial combination of pH treatments and Al concentrations. Variance for pH treatments (pH 4.5, 4.0, or 3.5) was partitioned into linear and quadratic components, and variance for Al concentrations (0, 13, 26 ppm) was also partitioned into the linear and quadratic components using orthogonal polynomial contrasts [22, 23]. For factors involved in a significant interaction, an examination of the average of treatment overall factors can be misleading [22]. Therefore, the response to Al was examined for each pH treatment using orthogonal contrast. An orthogonal contrast using each fixed level of pH treatment across either linear or quadratic components of Al treatments was developed to determine if pH treatment influenced the effect of Al treatments separately. A similar procedure of statistical analysis was conducted for responses to pH treatments.

For treatments of Ca/Al ratios at 26 ppm of Al, data were analyzed using Duncan's New Multiply-Range Test.

## Results

### Individual and combined effects of Al and low pH:

#### Visible foliar injury

Leaves of red pine seedlings grown in 26 ppm Al solution with pH 4.0 and 3.5 exhibited purplish leaves, which is similar to that of phosphorus deficiency, within 2 weeks after the initiation of the Al treatments. The seedlings grown in the solution of pH 3.5 containing Al at 13 ppm also slightly exhibited purplish leaves at the same time. However, there was no visible foliar injury at pH 4.5 over all the levels of Al.

#### Needle elongation

**Fig. 1:**
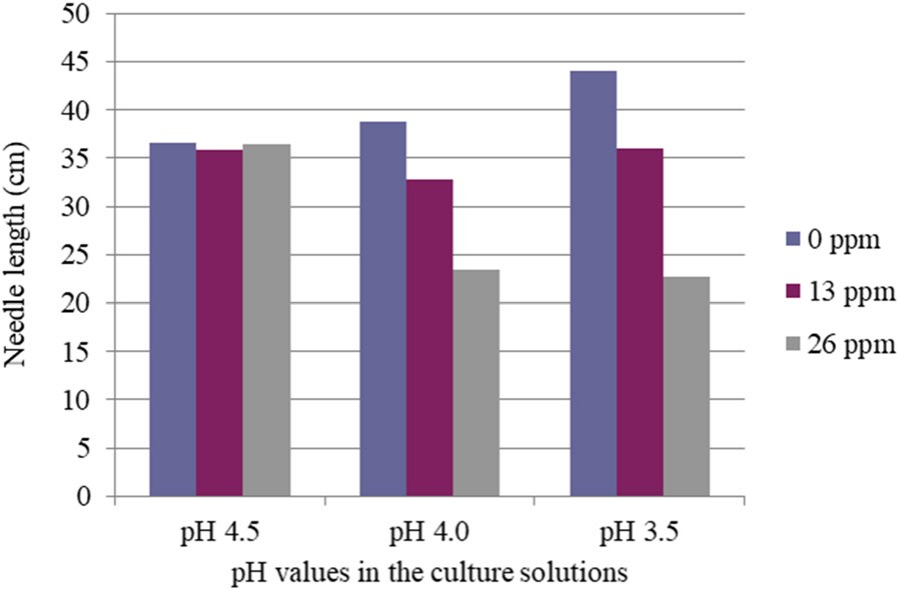
Single and interactive effects of low pH and high Al concentrations on needle length (cm)

The interactive effects of Al concentrations and low pH were significant and linearly synergistic (Table 2). The Al toxicity to current needle elongation was increased by reducing solution pH. In pH 4.5 treatment, Al had no toxic effects on the needle elongation, but in pH 4.0 or pH 3.5 treatment, needle elongation was linearly decreased with rising Al concentrations. On the other hand, Al toxicity at 26 ppm to the needle elongation was linearly enlarged with a reducing pH from 4.5, via 4.0, to 3.5. Without Al, the effects of solution pH values alone on needle elongation were not significant (Fig. 1, Table 2).

**Table 2:** Mean squares and levels of significance of Al and low pH in a culture solution for whole plant dry weight, needles dry weight, and needle length

Source of variation	D.F	Mean squares^a^		
		Whole plant	Needle	Needle
		DW	DW	Length
Al treatment	2	0.370*	0.148*	22.975**
Linear (Lin)	1	0.325**	0.147**	20.700***
Quadratic (Q)	1	0.045	0.001	0.275
pH treatment 2	2	0.055	0.022	2.881
Lin	1	0.051	0.002	0.544
Q	1	0.004	0.020	2.337
Al × pH	4	0.275	0.061	11.333^+^
Lin × lin	1	0.069	0.039^+^	10.350**
Lin × Q	1	0.016	0.001	0.647
Q × lin	1	0.179^+^	0.010	0.320
Q × Q	1	0.026	0.011	0.016
Error	99	5.093	1.194	137.919

^a^Calculated F-values significant at 0.10, 0.05, 0.01, or 0.001 levels are denoted by ^+, *, **^, or ^***^, respectively.

#### Dry weights

**Fig. 2:**
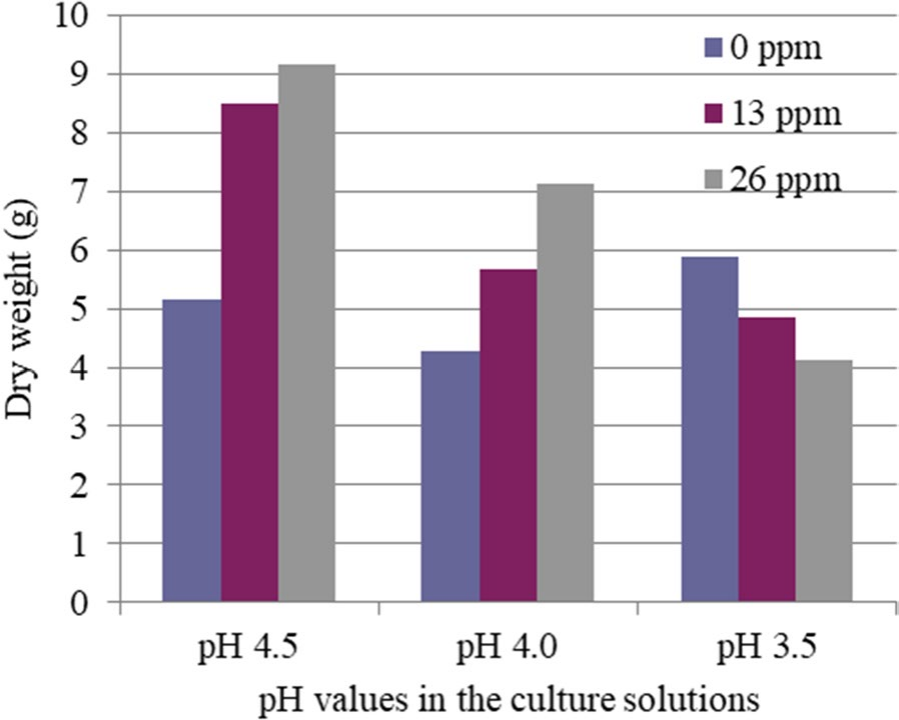
Single and interactive effects of low pH and high Al concentrations on the whole plant dry weight (g).

Needle, stem, and root dry weights of red pine seedlings were measured at the final harvest. Synergistic interactive effects of Al concentrations and pH in the whole plant dry weight were significant (Table 2). In pH 4.5 or 4.0 treatment, Al concentrations did not significantly affect whole plant dry weight. With reducing pH, Al toxicity was increased. Whole-plant dry weight was decreased linearly with increasing Al concentration in pH 3.5 treatment (Fig. 2, Table 2).

**Fig. 3:**
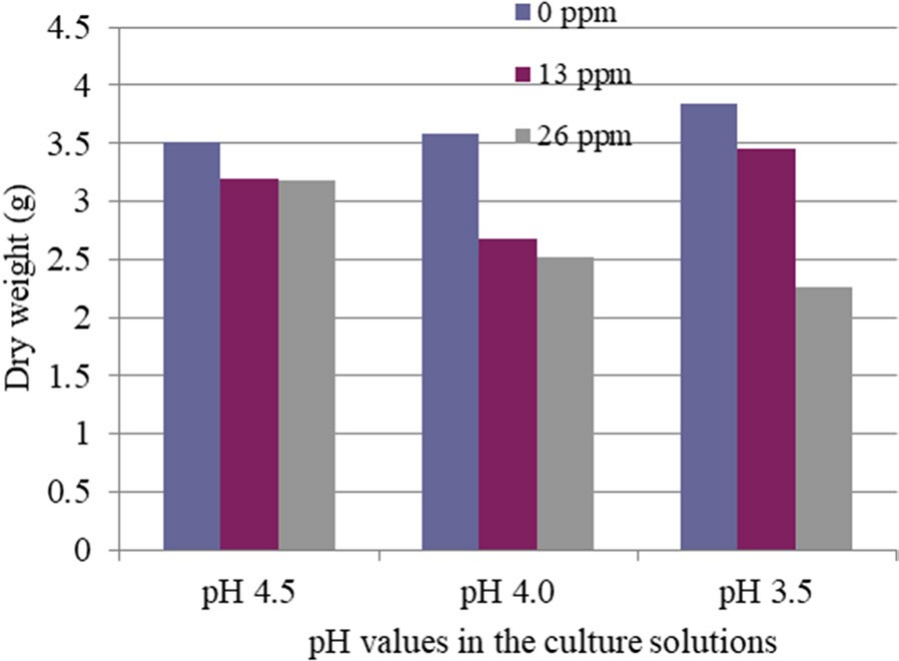
Single and interactive effects of low pH and high Al concentrations on needle dry weight (g)

Responses of needle dry weight to Al and low pH were similar to those of needle elongation (Table 2 and Fig. 3). However, no significant effects of pH and Al concentrations on the stem and root weights were observed (data not shown here).

Without Al, pH values in the culture solutions alone did not significantly affect all the parameters measured here (Figs. 1, 2, 3).

### Effects of Ca/Al molar ratio on al phytotoxicity

Root length and whole plant length were significantly decreased at Ca/Al molar ratios of 1:10 with 26 ppm Al compared with Ca/Al molar ratios of 1:1 or 10:1 (Table 3), but insignificantly changed for root, stem, needle dry weights, and whole plant fresh weights. The Ca concentrations in the solution cultures without Al did not affect the root length and whole plant length of red pine seedlings (data not shown here).

**Table 3:** Effects of Calcium/Aluminum (Ca/Al) molar ratios on phytotoxicity of Al when Al concentration was 26 pm

Parameters	Calcium/Aluminum ratios		
	10:1	1:1	0.1:1
Total fresh weight (g)	2.39^a^	1.80^a^	1.67^a^
Root length (cm)	18.10^a^	20.90^a^	12.70^b^
Whole-plant length (cm)	29.60^a^	28.00^a^	19.60^b^

The different letters of a or b beside the average values show a significant difference at p < 0.05 according to Duncan's New Multiply-Range Test.

## Discussions

Purplish leaves were observed in red pine seedlings grown in the solutions containing Al with pH 4.0 or 3.5 in this study. Red spruce, white spruce, and black spruce treated with phytotoxic levels of Al in solution culture exhibited brownish or blackened root systems and yellowish or purplish needles characteristics [2]. These observations are consistent.

The root of the plant is the most easily affected organ by Al toxicity. Al toxicity is more readily characterized by root morphology [4]. Root length, rate of root elongation, and weight of plant tops are reliable measures of Al toxicity, but root weight is not [24–28]. In the present study, we observed that needle elongation, needle dry weight, and especially root length and whole plant length are significantly reduced due to Al toxicity, but not stem dry weight and root dry weight. These results were consistent. Therefore, we focused on root and whole length and investigated the single and interactive effects of Al and Ca/Al ratio concentration in the second part of our experiments. No significant differences in the growth of red pine seedlings between pH 4.5, 4.0, or 3.5 without Al in solution cultures were observed. Our results showed that red pine could tolerate acidic conditions and is relatively tolerant to low pH. However, the synergistic interaction of low pH and the elevated Al concentrations was significant with the reduction in current needle length, and whole plant and needle dry weights (Table 2, Figures. 1, 2, 3). These results indicate that Al toxicity is significantly increased with reduced pH. It suggests that soil pH reduction caused by acid deposition or other factors can result in increases in not only Al concentration but also Al toxicity. It was well documented that plant growth suppression in low pH soils is mainly a result of Al toxicity rather than H^+^ toxicity except for extreme cases[29–31]. Our results showed that high Al concentration was more closely related to the growth reduction induced by soil acidification than low soil pH. The growth reduction of red pine seedlings was caused by the synergistic interaction of high Al concentration and low pH. Photosynthetic rates and growth of woody plants have been inhibited by elevating Al concentrations at low pH [7, 32]. In the earth’s crust, Al is the most abundant metallic element. The Al released from soil minerals under acidic conditions exists as Al(OH)_2_ + , Al(OH)_3_ + , and Al(H_2_O)_3_ + , which commonly affect Al toxicity [33]. As a result, low pH often becomes a problem with high Al concentrations. These findings suggest that red pine may exhibit similar growth reductions in acid soils with comparable Al concentration and low pH. The synergistic interaction of high Al concentration and low pH further deteriorated by anthropogenic input from acid deposition has adverse effects on the health of red pine forests. Six experimental forests in northwestern Germany were monitored in long-term studies. Although internal natural sources are relevant contributors to the total proton load, at five of the six sites, the H^+^ load due to atmospheric deposition exceeds 70% of the total proton load [15]. Therefore, acid deposition plays a major role in recent forest declines of red pine and other forests and crop production reduction caused by soil acidification problems in areas polluted by acid deposition in Europe, North America, and Asia.

Plants can be grouped into three general classes regarding their sensitivity to aluminum [20]: sensitive species injured by Al at concentrations between 25 and 250 mmol/m^3^, such as clovers [34, 35], intermediate species showing injuries between 500 and 1000 mmol/m^3^, such as peach seedlings [36], and insensitive plants not injured more than 3000 mmol/m^3^, such as certain conifers [37]. Our study demonstrated that the toxic threshold for Al injury to red pine seedlings appears to be between 370 and 1111 mmol/m^3^. Therefore, red pine is an intermediate species in sensitivity to Al.

This solution culture experiment showed that Al phytotoxicity also varied with Ca/Al ratio and was reduced with elevated Ca/Al ratio. It showed that root length and whole plant length were decreased with reducing Ca/Al ratios at 26 ppm of Al (Table 3). It has been reported that Al toxicity in forest trees will be manifested with a Ca/Al molar ratio < 1 [38] or < 2 [39]. The Ca effect cannot be explained in terms of the alleviation of Ca deficiency because Ca concentration elevated from 0.3 to 3 or 30 mM without Al did not increase root length and whole plant length of red pine seedlings in this study. Farmers in many nations worldwide have historically applied lime to acid soil to raise crop production. Such a favorable measure is due to not only raised soil pH but also reduced dissolved Al concentration, elevated Ca/Al ratio, and reduced phytotoxicity of Al. These results also suggest that liming is still an applicable measure to cope with soil acidification problems induced by natural factors such as rain leaching or anthropogenic factors such as acid depositions.

## Conclusion

The present results of solution culture experiments illustrate that the interactive effects of Al and low pH values are synergistic. Red pine is an intermediate species in sensitivity to Al toxicity. Al toxicity to red pine seedlings decreases with Ca/Al ratios increasing. Liming is still an applicable measure to cope with soil acidification problems induced by natural factors such as rain leaching or modern industry pollutions such as acid depositions.

## Data Availability

The datasets generated during and/or analyzed during the current study are available from the corresponding author on reasonable request.
